# Raman spectroscopy and topological machine learning for cancer grading

**DOI:** 10.1038/s41598-023-34457-5

**Published:** 2023-05-04

**Authors:** Francesco Conti, Mario D’Acunto, Claudia Caudai, Sara Colantonio, Raffaele Gaeta, Davide Moroni, Maria Antonietta Pascali

**Affiliations:** 1 Institute of Information Science and Technologies, National Research Council of Italy, Via G. Moruzzi 1, Pisa, 56124 Italy; 2 Department of Mathematics, University of Pisa, Largo B. Pontecorvo, 56126 Pisa, Italy; 3 Institute of Biophysics, National Research Council of Italy, Via G. Moruzzi 1, 56124 Pisa, Italy; 4 Division of Surgical Pathology, Department of Surgical, Medical, Molecular Pathology and Critical Area, University of Pisa, Via Paradisa 2, 56124 Pisa, Italy

**Keywords:** Biomedical engineering, Applied mathematics, Bone cancer, Biophysics

## Abstract

In the last decade, Raman Spectroscopy is establishing itself as a highly promising technique for the classification of tumour tissues as it allows to obtain the biochemical maps of the tissues under investigation, making it possible to observe changes among different tissues in terms of biochemical constituents (proteins, lipid structures, DNA, vitamins, and so on). In this paper, we aim to show that techniques emerging from the cross-fertilization of persistent homology and machine learning can support the classification of Raman spectra extracted from cancerous tissues for tumour grading. In more detail, topological features of Raman spectra and machine learning classifiers are trained in combination as an automatic classification pipeline in order to select the best-performing pair. The case study is the grading of chondrosarcoma in four classes: cross and leave-one-patient-out validations have been used to assess the classification accuracy of the method. The binary classification achieves a validation accuracy of 81% and a test accuracy of 90%. Moreover, the test dataset has been collected at a different time and with different equipment. Such results are achieved by a support vector classifier trained with the Betti Curve representation of the topological features extracted from the Raman spectra, and are excellent compared with the existing literature. The added value of such results is that the model for the prediction of the chondrosarcoma grading could easily be implemented in clinical practice, possibly integrated into the acquisition system.

## Introduction

Raman spectroscopy (RS) is a noninvasive optical technique sensitive to the molecular composition of biological tissues so that RS can be used to optically probe the molecular changes associated with disease tissues, making it possible to classify malignant cancer degrees^1^. Raman spectrum is a plot of scattered intensity as a function of the energy difference between the incident and scattered photons and is obtained by pointing a monochromatic laser beam at the tissue under investigation. Hence, the loss or gain in the photon energies corresponds to the difference in the final and initial vibrational energy levels of the molecules belonging to the specific spots of the tissue investigated. The difference between final and initial vibrational energy levels denote shifts in wavenumbers, which are unique for individual molecules resulting in specific peaks that are spectrally narrow and potentially associated with the vibration of a specific chemical bond in the molecules^2^.

Since the grading of cancer tissues is one of the main challenges for pathologists, RS is establishing itself as one of the most promising new techniques for supporting pathologists in making diagnoses as accurate as possible, avoiding or limiting as much as possible false positives and false negatives, unfortunately still commonly experienced by pathologists today, and increasing the overall accuracy of diagnostic protocols^3–7^. Recently, RS has been applied to chondrogenic tumour classification with excellent results^8^. Chondrogenic tumours are the second worldwide largest group of bone tumours, whose histologic pattern suggests a deep relationship to hyaline cartilage. Chondrosarcomas are tumours whose malignant cells produce a cartilaginous matrix. When they occur in previously normal bones, they are generally classified as primary chondrosarcomas. At the same time, secondary chondrosarcomas result from the malignant transformation of a benign cartilaginous lesion. They are classified into three malignant degrees, the first degree (CS G1), the second one (CS G2) and the third one (CS G3). In addition to such three degrees, Enchondroma (EC) is a noncancerous version. Distinguishing between EC and CS G1 is a rather critical issue for pathologists, generating many false positive and false negative diagnoses^9,10^. In order to adequately address the solution to this problem, RS has proved extremely useful^8^. Multivariate analysis is the basic discriminant approach able to handle Raman data to perform a diagnosis. The first application of multivariate statistics to chondrosarcoma has been primarily based on the principal component analysis—linear discriminant analysis (PCA-LDA) algorithm together with leave-one-out cross-validation method, yielding the sensitivities of 70% between EC and G1, and 90%, between G1 and G2, respectively. These results have indicated that Raman spectroscopy combined with multivariate analysis techniques can be used to explore the biochemical intravariability of the cancerous tissue under investigation^8^. A more recent paper^11^ exploited a more complex processing scheme, i.e. CLARA (CLAssification through wavelet transform of RAman spectra). CLARA is a two-stage classification method: the first stage directly uses the 1D signal to discriminate between EC, CS G1 and CS G2, G3, while the second stage applies the wavelet transform to Raman spectra in order to discriminate between EC and CS G1. CLARA achieves a 97% accuracy in the 3-label classification.

In this paper, we propose a novel method leveraging the topological features extracted from the Raman spectrum, to enhance the classification capability of standard machine learning techniques in classification. Even if the experimental dataset is not large, results show that such a method outputs a classification model which not only achieves high accuracy on never before seen data samples but also can be easily integrated into a Raman spectroscopic system as an automatic tool for supporting clinicians in grading the tumour.

The following section is devoted to the description of both the experimental data (Dataset 1 and Dataset 2) and the processing pipeline. In “Results” section, the processing pipeline is applied to the experimental data, and the classification results are reported; also, this section includes two ablation studies showing that the pipeline is more efficient than using only topological data analysis or only machine learning classifiers. The section closes with: (i) a thorough comparison of the best result we found in the state of the art; (ii) a description of the results achieved on new data (which have been acquired on new subjects using a different acquisition system). “Final test with new data” section discusses the best results achieved and concludes the paper.

## Materials and methods

### Data acquisition

**Figure 1:**
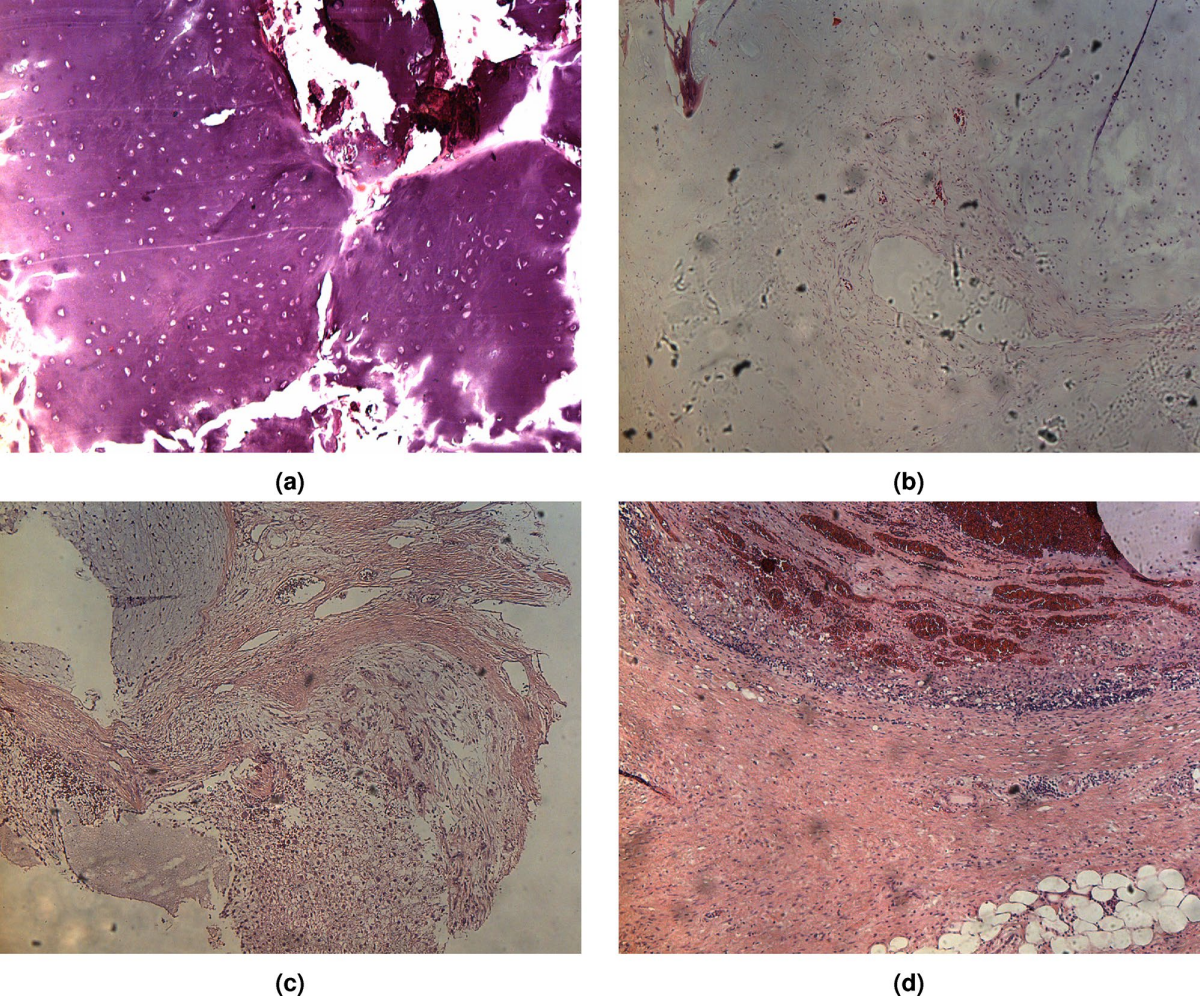
Representative histologic images of the tumours analyzed in this study (hematoxylin and eosin staining). EC (**a**); CS G1 (**b**); CS G2 (**c**); CS G3 (**d**).

**Figure 2:**
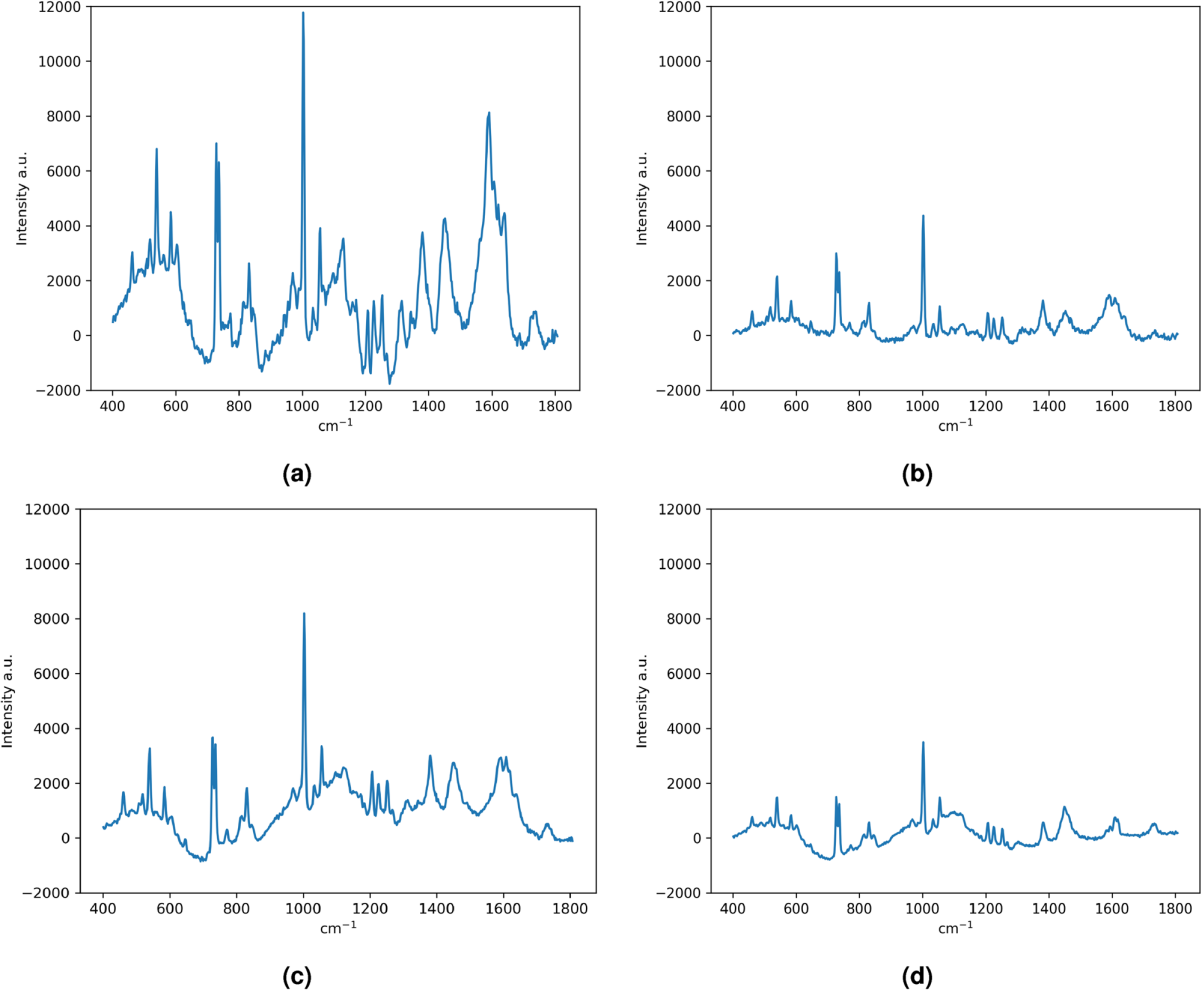
Representative Raman spectra of the tumors analyzed in this study. EC (**a**); CS G1 (**b**); CS G2 (**c**); CS G3 (**d**).

**Figure 3:**
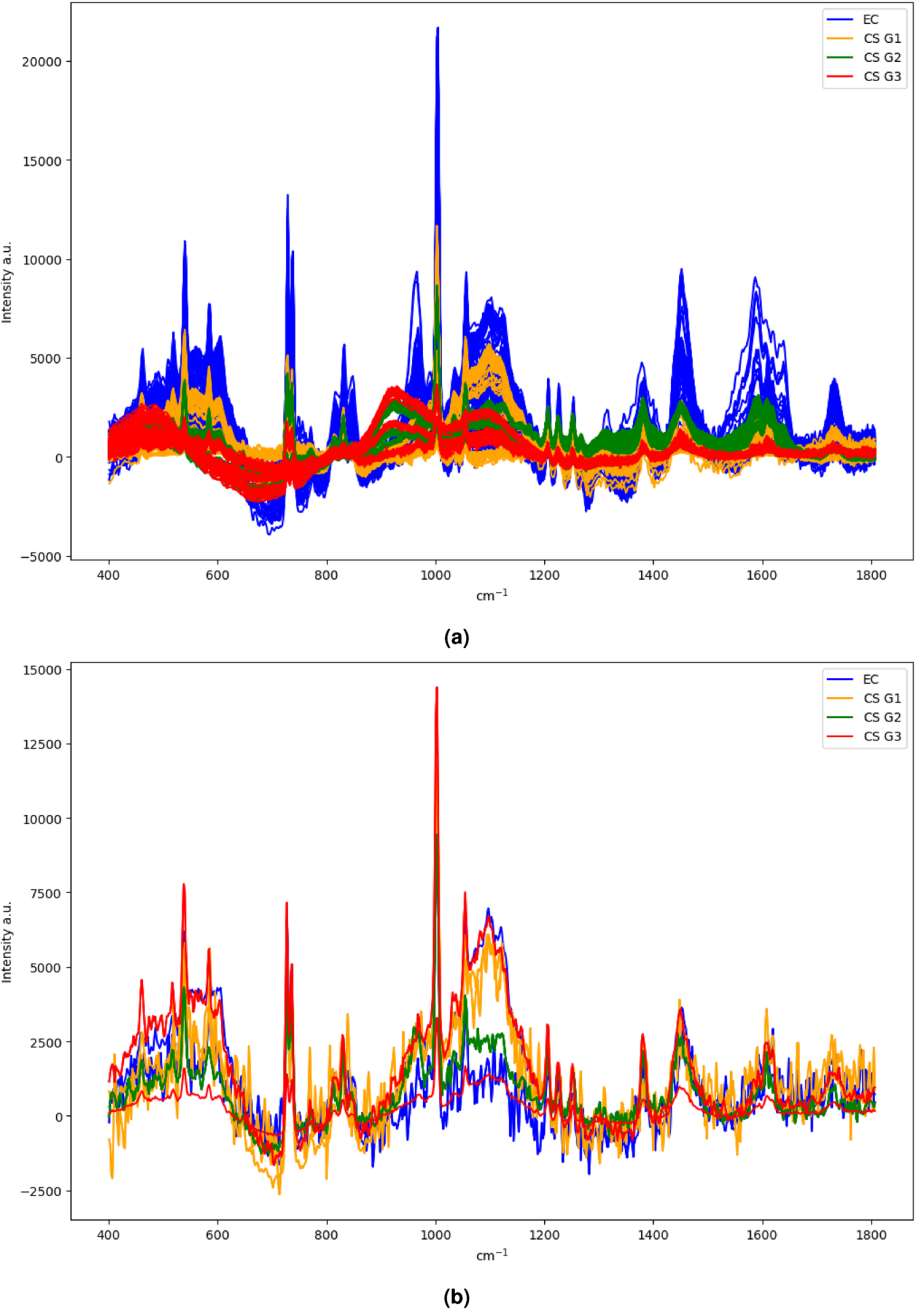
The entirety of Raman spectra coming from Dataset 1 (**a**) and from Dataset 2 (**b**).

The data acquisition was carried out with a Thermo Fisher Scientific DXR2xi Raman microscope. A total of 10 patients, who were being treated at the Institution, Azienda Ospedaliera Universitaria Pisana, Pisa, were enrolled in the study under the Ethical Committee agreement. Details can be found in the paper^8^. Formalin-fixed paraffin-embedded tumour tissue sections (e.g. in Fig. 1) were collected on glass slides and subsequently submitted to RS analysis after the dewaxing step (e.g. in Fig. 2). The protocol to remove paraffin and formalin has provided the immersion of the histopathological sections in a series of two baths of xylene for 10 min, respectively, and then washing the sections in PolyButylene Succinate (PBS) to remove residual formalin. Moreover, to give an idea of the variability of the datasets, Fig. 3 shows the Raman spectra coming from Dataset 1 (Fig. 3a) and from Dataset 2 (Fig. 3b).

The Raman spectroscopy measurements were configured based on the following experimental parameters: laser wavelength 532 nm; power laser of 5–10 mW; 400–3400 cm$^{-1}$ full range grating; 10×, 50× and 100× objectives; 25 µm pinhole; 5 (FWHM) cm$^{-1}$ spectral resolution. Integration time for recording a Raman spectrum was 1 s and 10 scans for any spectrum. As a first step, the tissue morphology overview was carried out to identify the regions of interest with the collection of a number of mosaic images at low (10×) and intermediate (50×) magnification. Thus, the acquisition of Raman spectra was carried out with a 100× objective. Optimization of signal-to-noise ratio and minimization of sample fluorescence were obtained through preliminary measurements in order to set the best experimental parameters. Multiple measurements were performed in different regions within the various samples, in order to assess intra-sample variability. In turn, no pre-treatment of the samples was necessary before Raman measurements. Minimal preprocessing, including background removal and baseline application, was performed using the tools of the DXR2xi GUI, and a $5{\text{th}}$ order polynomial correction was used to compensate for the tissue fluorescence. Peaks were identified with specific tool support by Omicron 9.0 software.

Raman hyperspectral chemical maps ranging from $50 \times 50\,\upmu \hbox {m}^2$ (step size 1 $\upmu \hbox {m}$) to approximately $200 \times 200\,\upmu \hbox {m}^{2}$ (step size $4\, \upmu \hbox {m}$), recording several hundreds of spectra per map were collected. Raman maps provide the fundamental advantage of being able to localize Raman spectra to specific locations, providing local information about chemical composition. Step sizes were chosen to have a collection time for each map less than 7 h for all the maps.

Ten supplemental spectra have been acquired, making use of an Xplora Plus (Horiba) in a similar experimental setup and preprocessing procedure in order to test the classification method on never seen data samples. This way, the results of the final test reported at the end of the “Results” section show that the classification method proposed is neither subject-dependent nor vendor-specific (DXR Thermo Fisher data for model training, Xplora Horiba data for final model testing).

In this paper, we have introduced the following labels for the machine learning part: EC = 0, CS G1 = 1, CS G2 = 2, CS G3 = 3. In the following, they will be used as synonyms. Finally, the data acquired have been split into two datasets:

- *Dataset 1* 400 spectra from ten subjects, belonging to the following chondrosarcoma malignancy classes [0, 0, 0, 1, 1, 1, 2, 2, 3, 3]. Each subject has respectively the following number of spectra: [32, 31, 37, 24, 38, 38, 50, 50, 49, 51].
- *Dataset 2* 10 spectra from ten subjects (no intersection with Dataset 1), belonging to the following chondrosarcoma malignancy classes [1, 2, 2, 1, 2, 0, 3, 3, 0].

### Data analysis

The core idea of our study is to employ the many tools of Topological Data Analysis (TDA) and machine learning (ML) to perform classification in the dataset of Raman spectra described in the previous section. The concept of using topological and geometrical ideas in medical data is not a novel one and has already demonstrated substantial potential through multiple research papers^12–18^.

In our approach, we evaluated the effectiveness of topological features by combining them with established machine learning algorithms, including support vector classifiers, random forest classifiers, and Ridge regressions. The reason why we preferred general machine learning algorithms to deep learning is twofold. Firstly, NNs are highly task dependent, whereas the adopted processing pipeline^19^ based on TDA and ML is intended to be very general. Secondly, for completeness, a CNN was trained on the persistence images obtained in “Combining TDA and ML: the classification pipeline” section. Since the results were not at all satisfactory, this experiment was excluded from this work.

#### Mathematical background

**Figure 4:**
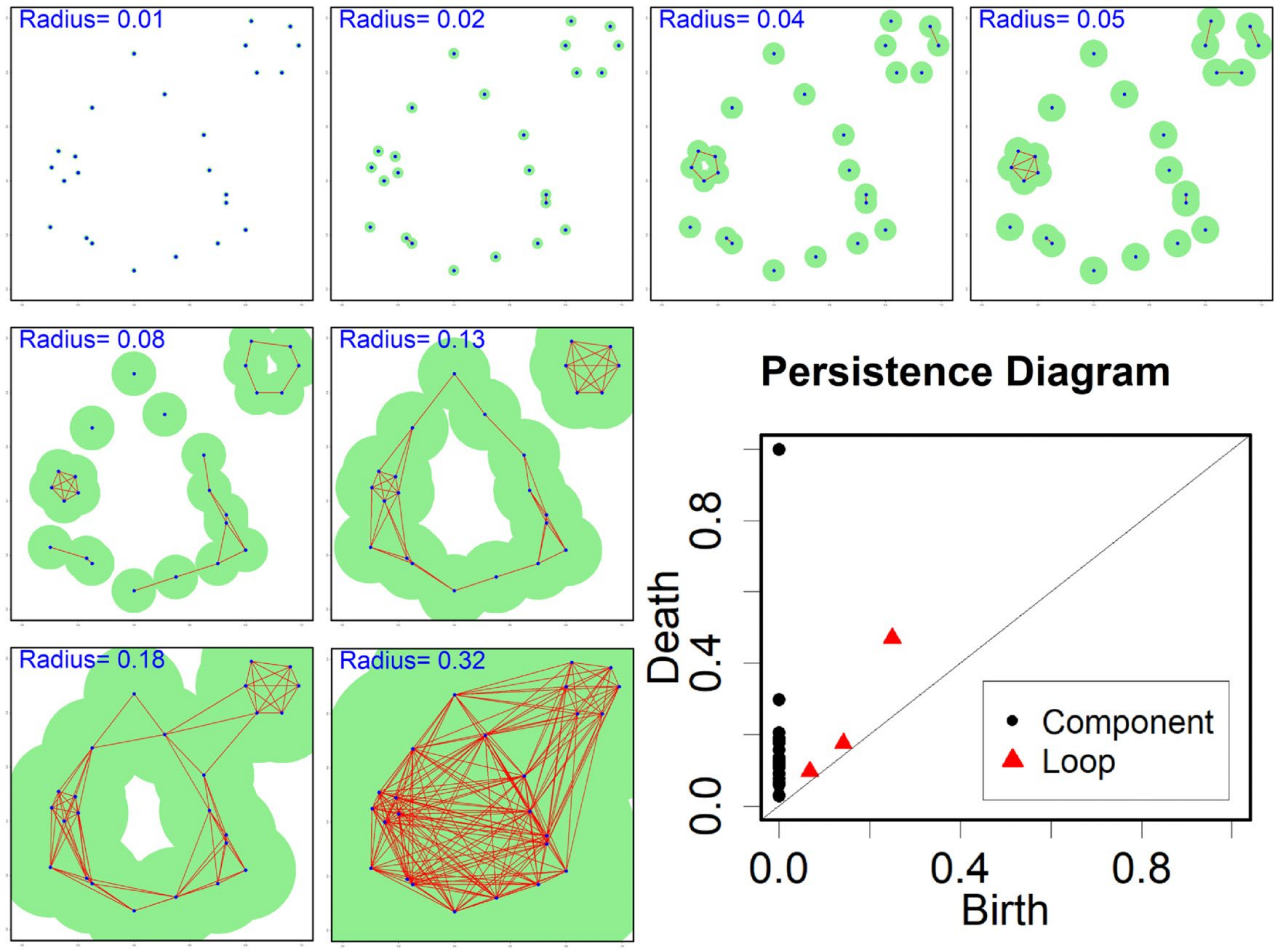
Data space at different scale resolutions (i.e. different radii) and the associated *k*-dimensional voids. The collection of such features forms the persistence diagram. Credits: Shafie Gholizadeh and Wlodek Zadrozny via A Tutorial on Topological Data Analysis in Text Mining^26^.

This section is mainly devoted to the description of TDA, a relatively new branch of applied mathematics that aims to bridge the gap between computational topology and discrete Morse theory in the study of high dimensional data. The interested reader can find more information about this topic here^20,21^. More precisely, we introduce Persistent Homology (PH) as one of the main concepts of TDA. Roughly speaking, PH studies the geometry of spaces by looking at the evolution of *k*-dimensional holes at different scales. It keeps track of the appearance and disappearance of such holes, which are the topological features, in the form of intervals $(\text {birth},\, \text {death})$. The persistence of a topological feature is the span of its detectability, and it is a measure of its importance. In particular, features with a longer lifespan are more likely to be key features in describing the shape of the data space, while features with a short lifespan can often be assimilated to noise. The collection of the intervals $(\text {birth},\, \text {death})$ is called the Persistence Diagram (PD). Mathematically, a persistence diagram is a multiset, which is a set where elements can appear multiple times, i.e. each element has a multiplicity. Different metrics can be defined in the space of persistence diagrams. Notwithstanding the precise mathematical definition of these metrics (for which we refer the reader to^20,21^), an essential property is that the process that associates a PD with data is stable with respect to these metrics. This means that a small perturbation in the data yields a small perturbation of the associated PD. This property is of fundamental importance in applications because it guarantees robustness against noise and repeatability. The main drawback of PDs is that the space of multisets lacks fundamental properties required in a machine learning context. For this reason, a number of representation methods have been devised in order to exploit the PDs’ expressiveness in ML algorithms. For more information on such representation methods, we refer the reader to these papers^22–25^. The key idea of all these methods is to embed the space of persistence diagrams in a more broad Hilbert space in a stable way, i.e. to *vectorize* the PD. After this last step, we are able to exploit the topological features extracted by persistent homology directly in a machine learning algorithm. Figure 4 shows the classical paradigm for topological data analysis.

#### Combining TDA and ML: the classification pipeline

**Figure 5:**
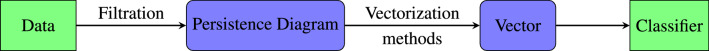
The pipeline for a topological study of digital data in a machine learning context. A filtration associates a persistence diagram with the digital data. The persistence diagram is then vectorized by means of various vectorization methods. Finally, the vector is fed to a machine learning classifier.

**Figure 6:**
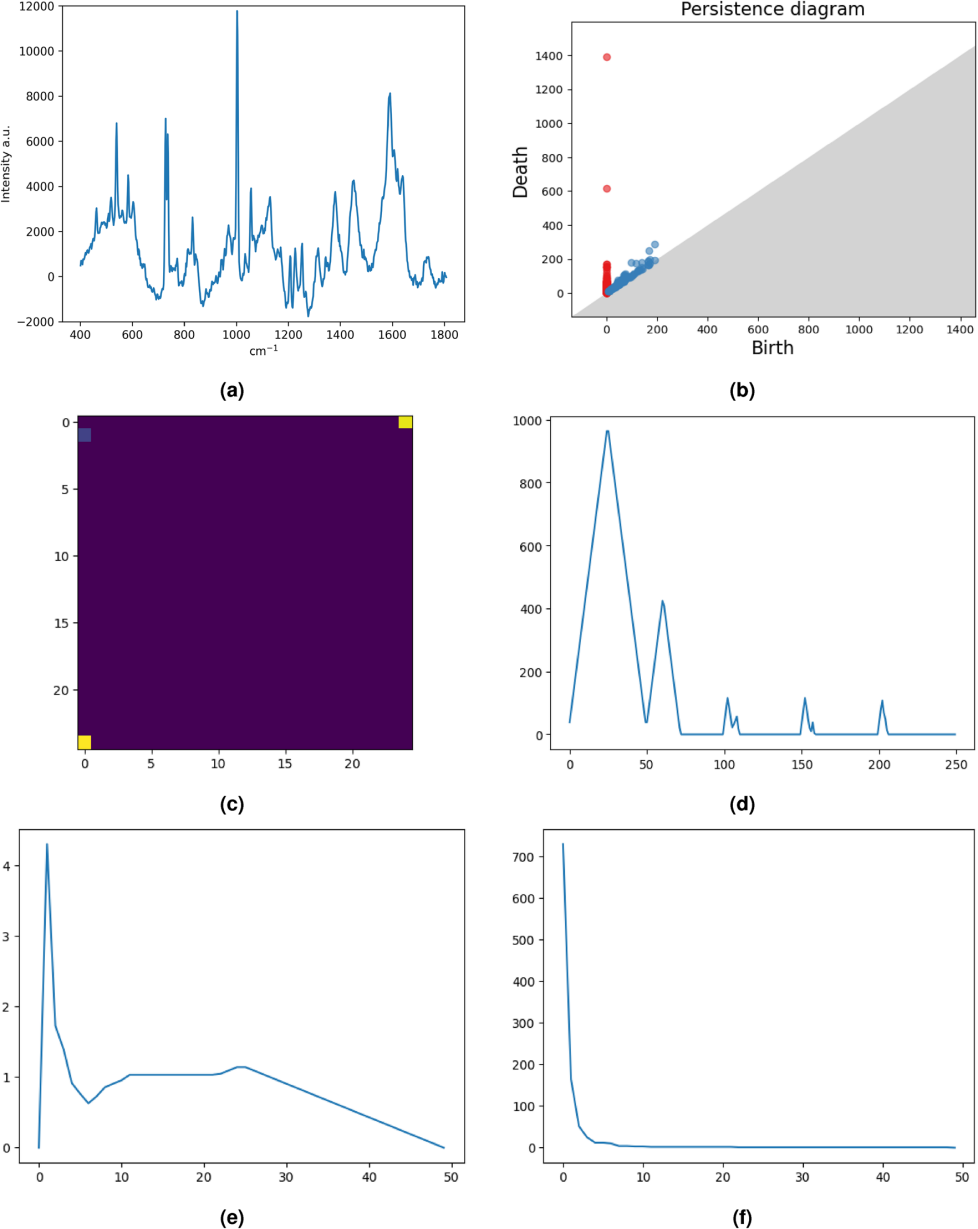
Pipeline application for the Raman spectra of chondrogenic tumours. The Raman spectra (**a**) and the persistence diagram associated (**b**). In the second and third rows, four different vectorization methods for the same PD, namely Persistence Image (**c**), Persistence Landscape (**d**), Persistence Silhouette (**e**) and Betti Curve (**f**).

This section is devoted to the description of the topological pipeline employed in this study. We refer to Fig. 5 for a general scheme of our approach. The classification pipeline consists of an automatic grid search for the optimal choice of (i) PH-base representation of the input data; (ii) ML classifier for cancer staging. Such a pipeline has already been presented in this article^19^, where a variety of tests on benchmark datasets were carried out. The present work describes its first application to experimental data. See Fig. 6 for a graphical example of our pipeline. The first step of the pipeline is to compute the PDs from the Raman Spectra. In doing so, we chose the Vietoris-Rips filtration^27^. In this approach, each point of the spectra is treated as a point in the Euclidean space $\mathbb {R}^2$. We grow balls centered at each point of the signal and when *i* balls intercept, an $i-1$ simplex is added to the simplicial complex with birth value *r*, the current radius. An alternative approach might have been to use lower star filtration^20,28^. Without going into details, since this filtration does not generate points in $H_1$ for 1*D* signals, Vietoris-Rips was preferred. Hence, starting from the Raman spectra (Fig. 6a), restricted to the wavenumber range 400–1800 $\text {cm}^{-1}$, the persistence diagram of homology in dimension 0 and 1 is computed with a Vietoris Rips filtration using the python Ripser package^29^ (Fig. 6b). The PD is therefore vectorized using four different vectorization methods with different combinations of parameters. More specifically, the PDs are vectorized using the following setup:

- Persistence Images^22^ (PI) with bandwidth $\sigma \in \{0.1, 1, 10\}$ and resolution $n \in \{5, 10, 25\}$ (Fig. 6c);
- Persistence Landscapes^23^ (PL) with resolution $n \in \{25, 50, 75, 100\}$ (Fig. 6d);
- Persistence Silhouette^24^ (PS) with resolution $n \in \{25, 50, 75, 100\}$ (Fig. 6e);
- Betti Curve^25^ (BC) with resolution $n \in \{25, 50, 75, 100\}$ (Fig. 6f).

It is important to highlight the fact that PDs produce points in different homological dimensions, and such information must be treated carefully. In more detail, following the rich TDA literature, we employed four different approaches to deal with information originating from different dimensions. In the first (resp. second) approach, only the points in dimension $H_i$ for $i = 0$ (resp. $i=1$) are considered in the vectorization. In the third one, the actual homology dimension is neglected, and all the points are vectorized altogether regardless of the dimension in which they show up. Finally, in the fourth approach, $H_0$ and $H_1$ are vectorized separately, and then the corresponding vectors are concatenated. We will refer to these approaches as $H_0$, $H_1$, $H_0 + H_1$ (fused) and $H_0 + H_1$ (concat) respectively. Such vectors represent the input for different machine learning classifiers. The classifiers employed in our pipeline are:

- Support Vector Classifier^30^ (SVC) with RBF kernel and $C \in \{1, 2, 3, 5, 10, 20\}$;
- Random Forest Classifier^31^ (RFC) with $\#\text {trees} = 100$;
- Ridge Regression^32^ (RR) with $\alpha = 1$.

These are well-known and standard ML classifiers; in this work, we used the implementation of the Scikit-learn library^33^. The pipeline performs a grid search between the four approaches, the different vectorization and classifiers and returns the accuracy of each method for each of the ten runs of a leave-one-patient-out cross-validation^34^ (LOPO). We stress that the design of our experimentation, including vectorizations, classifiers and LOPO, is motivated by two main reasons: (i) to achieve enough consistency with the previous work of the pipeline^19^ and others TDA papers; (ii) the limited amount of available data allows for meticulous research of optimality.

### Ethics statements

The study was approved by the local Ethical Committee *Comitato Etico Regionale per la Sperimentazione Clinica della Regione Toscana sezione AREA VASTA NORD OVEST* (protocol number 14249). Ten patients affected by primary chondrogenic tumours of the skeleton were enrolled in this study. Informed consent was collected from all patients. All the experiments were carried out in accordance with Good Clinical Practice (GCP) and with the ethical principles of the Declaration of Helsinki. All patients were diagnosed and treated at Azienda Ospedaliera Universitaria Pisana, Pisa, in 2018.

## Results

In this section, we are going to explore the results achieved by the pipeline described in “Combining TDA and ML: the classification pipeline” section. Due to the scarcity of data, we were able to perform a large number of experiments without any kind of computational restriction. For a more detailed description of the experimental data, please refer to “Data acquisition” section. In our first experiment (“Supervised results” section), we performed supervised learning on Dataset 1. In more detail, in “Supervised results” section we trained different combinations of labels. Specifically, we experimented by training the classifier with 4 labels (“LOPO validation 4 labels” section), with 3 labels (EC *vs.* CS G1 *vs.* CS G2 and CS G3, “LOPO validation 3 labels (EC vs. CS G1 vs. CS G2 and G3)” section) and two binary classifiers (EC *vs.* CS, “LOPO validation 2 labels (EC vs. CS)” section; EC and CS G1 *vs.* CS G2 and CS G3, “LOPO validation 2 labels (EC, CS G1 vs. CS G2, G3)” section). As explained in “Introduction” section, the most clinically meaningful subdivision is the binary classification EC *vs.* CS. Nevertheless, other subdivisions that may be clinically useful were also investigated, as a proof-of-concept study of the applicability of our method to the more challenging task of supporting the pathologist in the tumour grading, i.e. the subdivision no cancer *vs.* mild cancer *vs.* severe cancer in “LOPO validation 3 labels (EC vs. CS G1 vs. CS G2 and G3)” section. Indeed, the results obtained with these subdivisions, although of limited validity due to the small number of patients, encourage to enlarge the experimentation in order to validate our method further. Moreover, in “Unsupervised clustering” section we performed unsupervised learning (clustering) on Dataset 1. In “Comparison with CLARA^11^” section, we compare with the state of the art from the paper^11^. Finally, in “Final test with new data” section, the best model of “LOPO validation 2 labels (EC vs. CS)” section (supervised, 2-label classification), trained on Dataset 1, was tested on Dataset 2, in order to assess its generalization capability.

The pipeline takes the Raman spectra as input, computes the PDs by means of a Vietoris Rips filtration, vectorizes the PDs, and feeds such vectors to a machine learning classifier: basically, we start from a vector, and we end up with another vector of topological features. For this reason, in “Supervised learning without TDA” section, an ablation study has been carried out by feeding the Raman spectra directly to the machine learning classifiers, and comparing with the results achieved. We recall that the pipeline performs a grid search between a large number of methods {vectorization method, classifier}. Moreover, we treat separately the different homology dimensions discussed in “Combining TDA and ML: the classification pipeline” section. For this reason, in the following, for each experiment, we report both a table showing the best accuracy among all methods for each run of the LOPO validation and each homology dimension, as well as a table showing the best single method (as average accuracy) for each homology dimension.

### Supervised results

In the first, somewhat naive experiment, we split all the spectra in training and test, not requiring to have all the spectra from the same patient in the training or in the test set (and not in both). Soon after, we opted for a leave-one-patient-out cross-validation (LOPO) approach to prevent overfitting. Moreover, we did not always carry out a 4-class classification, but also conducted 3 and 2-class studies in accordance with the existing literature.

#### Tenfold cross-validation with 4 labels

The first experiment uses all the 400 spectra from Dataset 1 and performs a tenfold cross-validation. We highlight that, in doing so, spectra coming from the same patient can occur both in the train dataset and in the test dataset. We report the classification accuracy of each run and each homology dimension in Table 1, while Table 2 reports the single best method for each homology dimension. Clearly, the accuracy results are extremely satisfying and fully justify a study of Raman spectra for chondrosarcoma tumour’s degree of malignancy.

**Table 1:** Accuracy of the pipeline with 4 labels and a tenfold cross-validation approach.

Accuracy:	$H_0$	$H_1$	$H_0+H_1$ (fused)	$H_0+H_1$ (concat)
Run 1	0.925 (PI)	0.842 (PI)	0.933 (PS)	0.933 (PI)
Run 2	0.958 (PI)	0.875 (PI)	0.925 (PI)	0.958 (PI)
Run 3	0.975 (PS)	0.833 (PL)	0.975 (PS)	0.967 (PS)
Run 4	0.958 (PI)	0.842 (PI)	0.958 (PS)	0.950 (PI)
Run 5	0.967 (PI)	0.792 (PI)	0.958 (PS)	0.942 (PS)
Run 6	0.950 (PI)	0.875 (PI)	0.958 (PS)	0.950 (PI)
Run 7	0.983 (PS)	0.858 (PI)	0.975 (PS)	0.950 (PI)
Run 8	0.958 (PI)	0.875 (PI)	0.967 (PS)	0.950 (PI)
Run 9	0.942 (PS)	0.858 (PL)	0.967 (PS)	0.917 (PI)
Run 10	0.925 (PS)	0.808 (PI)	0.933 (PS)	0.942 (PI)
Mean:	$0.954 \pm 0.018$	$0.846 \pm 0.027$	$0.955 \pm 0.017$	$0.946 \pm 0.013$

**Table 2:** Best method with 4 labels and a tenfold cross-validation approach.

Homology	Accuracy	Vectorization	Classifier
$H_0$	0.943	PS	RFC
$H_1$	0.824	PI	SVC
$H_0+H_1$ (fused)	0.948	PS	RFC
$H_0+H_1$ (concat)	0.940	PI	RFC

#### LOPO validation 4 labels

In this second experiment, we repeat the same experiment of “Tenfold cross-validation with 4 labels” section but using a leave-one-patient-out cross-validation approach. That is, in turn, all spectra from the same patient were used as test and all other spectra from other subjects as train. Table 3 reports the accuracy results over the course of the ten runs of the leave-one-patient-out and Table 4 the best single method in terms of accuracy. Clearly, there is a huge difference with the results of the previous experiment. We want to focus on a very important aspect of these results. Regardless of the homology dimension considered, runs 1, 2, 3 and 6 obtain high accuracy, runs 4 and 5 fluctuating accuracy while the accuracy is low in the remaining runs. Comparing these runs with the composition of Dataset 1, described in “Data acquisition” section, we note that in the train dataset of runs 7, 8, 9, 10 there were spectra from a single patient, while in runs 4 and 5 there were spectra from two patients, but one in significantly greater numbers than the other, and finally in the remaining runs there were always spectra from two patients in the train set. These facts, together with the excellent results obtained in “Tenfold cross-validation with 4 labels” section, lead us to think that our method is effective in correctly classifying spectra, but the variability of spectra from a single patient is low and at least the spectra of two patients in the train set are needed to learn the class features. Otherwise, the classifier only learns to recognize the patient.

**Table 3:** Accuracy of the pipeline with 4 labels and a leave-one-patient-out cross-validation approach.

Accuracy	$H_0$	$H_1$	$H_0+H_1$ (fused)	$H_0+H_1$ (concat)
Run 1	0.844 (PI)	0.844 (PI)	0.844 (PI)	0.844 (PI)
Run 2	1.000 (PI)	1.000 (PI)	1.000 (PI)	1.000 (PI)
Run 3	0.973 (BC)	0.892 (PI)	1.000 (PI)	1.000 (BC)
Run 4	0.791 (BC)	0.333 (BC)	0.792 (BC)	0.792 (BC)
Run 5	0.447 (BC)	0.289 (PL)	0.421 (BC)	0.395 (PL)
Run 6	1.000 (PI)	0.868 (PI)	1.000 (BC)	1.000 (PI)
Run 7	0.100 (PI)	0.160 (PI)	0.160 (BC)	0.100 (PI)
Run 8	0.040 (PL)	0.300 (PL)	0.002 (PI)	0.100 (PL)
Run 9	0.204 (BC)	0.245 (BC)	0.204 (PI)	0.408 (BC)
Run 10	0.078 (PL)	0.294 (PS)	0.020 (PI)	0.255 (PL)
Mean:	$0.548 \pm 0.393$	$0.523 \pm 0.314$	$0.546 \pm 0.400$	$0.589 \pm 0.357$

**Table 4:** Best method with 4 labels and a leave-one-patient-out cross-validation approach.

Homology	Accuracy	Vectorization	Classifier
$H_0$	0.411	BC	SVC
$H_1$	0.300	PI	Ridge
$H_0+H_1$ (fused)	0.433	BC	SVC
$H_0+H_1$ (concat)	0.395	BC	SVC

#### LOPO validation 3 labels (EC *vs.* CS G1 *vs.* CS G2 and G3)

In our third experiment, we repeat a leave-one-patient-out approach but only with 3 labels, i.e., more specifically, class EC *vs.* CS G1 *vs.* CS G2 and CS G3. The results in Table 5 show a marked improvement over “LOPO validation 4 labels” section, but there is no stability in the method that achieves them; also, the accuracy of the best single method, shown in Table 6, is not as satisfactory.

**Table 5:** Accuracy of the pipeline with 3 labels and a leave-one-patient-out cross-validation approach.

Accuracy	$H_0$	$H_1$	$H_0+H_1$ (fused)	$H_0+H_1$ (concat)
Run 1	0.844 (PI)	0.813 (PI)	0.844 (PI)	0.844 (PI)
Run 2	0.973 (PI)	1.000 (PI)	1.000 (PI)	1.000 (PI)
Run 3	0.792 (BC)	0.622 (BC)	0.973 (BC)	1.000 (BC)
Run 4	0.421 (BC)	0.292 (PI)	0.792 (BC)	0.750 (BC)
Run 5	1.000 (BC)	0.316 (PL)	0.421 (BC)	0.447 (PL)
Run 6	1.000 (PI)	0.974 (PI)	0.974 (BC)	1.000 (PI)
Run 7	1.000 (PI)	1.000 (PI)	1.000 (PI)	1.000 (PI)
Run 8	1.000 (PI)	1.000 (PI)	0.960 (PI)	0.780 (PI)
Run 9	1.000 (PI)	1.000 (PI)	1.000 (PI)	1.000 (PI)
Run 10	1.000 (PI)	1.000 (PS)	1.000 (PI)	1.000 (PI)
Mean:	$0.902 \pm 0.176$	$0.802 \pm 0.275$	$0.896 \pm 0.173$	$0.882 \pm 0.174$

**Table 6:** Best method with 3 labels and a leave-one-patient-out cross-validation approach.

Homology	Accuracy	Vectorization	Classifier
$H_0$	0.586	PI	SVC
$H_1$	0.531	PI	SVC
$H_0+H_1$ (fused)	0.569	PI	SVC
$H_0+H_1$ (concat)	0.581	PI	SVC

#### LOPO validation 2 labels (EC *vs.* CS)

In this experiment, we again performed supervised learning with a leave-one-patient-out cross-validation approach with only two labels, specifically class EC versus CS, viz. the benign class versus the malignant ones. We highlight the fact that in this way the balance of classes is lost. In particular, the EC class represents only 25% of the dataset. We recall that in the literature this is the most meaningful subdividion. We report the accuracy results in Tables 7 and 8. The accuracy results are promising in both tables, being above 98% in the first one, and above 80% in the second one.

**Table 7:** Accuracy of the pipeline with 2 labels (EC *vs.* CS) and a leave-one-patient-out cross-validation approach.

Accuracy	$H_0$	$H_1$	$H_0+H_1$ (fused)	$H_0+H_1$ (concat)
Run 1	0.844 (PI)	0.750 (PI)	0.844 (PI)	0.844 (PI)
Run 2	1.000 (PI)	1.000 (PS)	1.000 (PI)	1.000 (PI)
Run 3	0.946 (BC)	0.595 (PL)	0.973 (BC)	0.973 (BC)
Run 4	1.000 (PI)	1.000 (PI)	1.000 (PI)	1.000 (PI)
Run 5	1.000 (BC)	1.000 (PI)	1.000 (PI)	1.000 (BC)
Run 6	1.000 (PI)	1.000 (PI)	1.000 (PI)	1.000 (PI)
Run 7	1.000 (PI)	1.000 (PI)	1.000 (PI)	1.000 (PI)
Run 8	1.000 (PI)	1.000 (PI)	1.000 (PI)	1.000 (PI)
Run 9	1.000 (PI)	1.000 (PI)	1.000 (PI)	1.000 (PI)
Run 10	1.000 (PI)	1.000 (PI)	1.000 (PI)	1.000 (PI)
Mean:	$0.978 \pm 0.048$	$0.934 \pm 0.136$	$0.982 \pm 0.047$	$0.982 \pm 0.047$

**Table 8:** Best method with 2 labels (EC *vs.* CS) and a leave-one-patient-out cross-validation approach.

Homology	Accuracy	Vectorization	Classifier
$H_0$	0.808	PI	SVC
$H_1$	0.799	PL	RFC
$H_0+H_1$ (fused)	0.814	BC	SVC
$H_0+H_1$ (concat)	0.810	PS	RFC

#### LOPO validation 2 labels (EC, CS G1 *vs.* CS G2, G3)

Finally, we repeat the experiment of “LOPO validation 2 labels (EC vs. CS)” section with different labels, that is classes EC, CS G1 *vs.* CS G2, G3, that is low/no malignancy versus high malignancy. The results are reported in Table 9 and Table 10, and again we obtain pretty good accuracies.

**Table 9:** Accuracy of the pipeline with 2 labels (EC, CS G1 *vs.* CS G2, G3) and a leave-one-patient-out cross-validation approach.

Accuracy	$H_0$	$H_1$	$H_0+H_1$ (fused)	$H_0+H_1$ (concat)
Run 1	1.000 (PS)	0.906 (PI)	1.000 (PI)	0.969 (BC)
Run 2	1.000 (PI)	1.000 (PI)	1.000 (PI)	1.000 (PI)
Run 3	1.000 (BC)	1.000 (PL)	1.000 (PI)	1.000 (BC)
Run 4	1.000 (BC)	0.875 (PI)	1.000 (PI)	0.958 (BC)
Run 5	1.000 (PI)	0.947 (BC)	1.000 (PI)	1.000 (PI)
Run 6	1.000 (PI)	0.526 (PI)	1.000 (PI)	1.000 (PI)
Run 7	1.000 (PI)	1.000 (PI)	1.000 (PI)	1.000 (PI)
Run 8	0.260 (PI)	0.620 (PL)	0.300 (PL)	0.380 (PL)
Run 9	1.000 (PI)	1.000 (PI)	1.000 (PI)	1.000 (PI)
Run 10	0.980 (PI)	1.000 (PS)	0.922 (PI)	1.000 (PI)
Mean:	$0.924 \pm 0.221$	$0.887 \pm 0.164$	$0.922 \pm 0.201$	$0.931 \pm 0.184$

**Table 10:** Best method with 2 labels (EC, CS G1 *vs.* CS G2, G3) and a leave-one-patient-out cross-validation approach.

Homology	Accuracy	Vectorization	Classifier
$H_0$	0.772	BC	SVC
$H_1$	0.669	BC	SVC
$H_0+H_1$ (fused)	0.763	BC	SVC
$H_0+H_1$ (concat)	0.770	BC	SVC

#### Supervised learning without TDA

As specified in “Results” section, our pipeline transforms vectors (Raman spectra) into vectors (topological features) before feeding them into a machine learning classifier. Therefore, to justify a topological study of Raman spectra, we perform an ablation study: in this experiment, we directly feed the Raman spectra in the same classifiers as in the pipeline and compute the accuracy following the same validation scheme. For the sake of brevity, we only report the mean accuracy and best classifier for each label classifier. We report these results in Table 11. We can see from the results that the accuracies are slightly worse. Moreover, it is clear how insignificant these results are, e.g., by looking at the case with 4 labels, where we have a variance greater than the accuracy value itself. Hence, we conclude that TDA increases both accuracy and, notably, consistency.

**Table 11:** Mean accuracy and best classifier accuracy for supervised learning without TDA.

Experiment	4 labels	3 labels	EC *vs.* CS	EC, CS G1 *vs.* CS G2, G3
Mean accuracy	$0.377 \pm 0.429$	$0.617 \pm 0.393$	$0.922 \pm 0.138$	$0.859 \pm 0.288$
Best classifier	0.286 (SVC)	0.494 (SVC)	0.843 (SVC)	0.791 (SVC)

### Unsupervised clustering

For completeness, we also performed an unsupervised study on the vectors obtained from the pipeline. That is, instead of feeding the vectorizations of the PDs to a machine learning classifier, we used them as input to a clustering algorithm. Following the extensive machine learning literature, we used the following clustering algorithms, all from the Scikit-learn library: Affinity propagation^35^, Agglomerative clustering^36^, BIRCH^37^, DBSCAN^38^, K-Means^39^, Mini batch K-Means^40^, Mean-shift^41^, OPTICS^42^, Spectral clustering^43^ and Gaussian mixtures^44^. We report the accuracy results for the experiment with 4 labels, 3 labels, 2 labels with class EC *vs.* CS and 2 labels with classes EC, CS G1 *vs.* CS G2, G3 in Tables 12, 13, 14 and 15 respectively. The most interesting aspect of these results is that decreasing in the number of labels does not produce an increase in accuracy, suggesting that indeed the features extracted by the TDA are sufficiently separated.

**Table 12:** Clusterization accuracy of the vectorized PDs with 4 labels.

Homology	Accuracy	Vectorization	Cluster
$H_0$	0.512	PS	Affinity propagation
$H_1$	0.400	PI	Gaussian mixture
$H_0+H_1$ (fused)	0.562	PS	Affinity propagation
$H_0+H_1$ (concat)	0.523	PS	Affinity propagation

**Table 13:** Clusterization accuracy of the vectorized PDs with 3 labels.

Homology	Accuracy	Vectorization	Cluster
$H_0$	0.458	PS	BIRCH
$H_1$	0.330	PI	Affinity propagation
$H_0+H_1$ (fused)	0.487	PS	Affinity propagation
$H_0+H_1$ (concat)	0.462	PS	BIRCH

**Table 14:** Clusterization accuracy of the vectorized PDs with 2 labels (EC *vs.* CS).

Homology	Accuracy	Vectorization	Cluster
$H_0$	0.501	PS	BIRCH
$H_1$	0.338	PI	DBSCAN
$H_0+H_1$ (fused)	0.487	PS	Mini batch K-Means
$H_0+H_1$ (concat)	0.487	PS	BIRCH

**Table 15:** Clusterization accuracy of the vectorized PDs with 2 labels (EC, CS G1 *vs.* CS G2, G3).

Homology	Accuracy	Vectorization	Cluster
$H_0$	0.464	PS	BIRCH
$H_1$	0.256	PS	Mean-shift
$H_0+H_1$ (fused)	0.399	PS	Mini batch K-Means
$H_0+H_1$ (concat)	0.459	PS	BIRCH

### Comparison with CLARA^11^

“Introduction” section highlights that the results we should compare to those described in the papers^8,11^, which achieve remarkable performances in classifying the same 400 Raman spectra. In details, CLARA is the best-performing method to achieve the 3-label classification. For a fair comparison, we applied the same preprocessing steps as in the paper^11^. Namely, we performed a data augmentation on Dataset 1 and chose a specific train-test split. The steps are described below.

#### Data augmentation

Following the paper^11^, Dataset 1 is augmented with the addition of some shifts in the spectra to simulate potential inaccuracies in the wavelength calibrations and with the addition of an additive noise sampled from a normal distribution. More specifically, each original spectrum is passed through:

- additive Gaussian noise with mean 0 and standard deviation (std) $\max (\text {spectrum})/1000$;
- additive Gaussian noise with mean 0 and std 1;
- shift uniform noise in the interval $[- 5, 5]$;
- additive Gaussian noise with mean 0 and std $\max (\text {spectrum})/1000$ and shift uniform noise in the interval $[- 5, 5]$;
- additive Gaussian noise with mean 0 and std 1 and shift uniform noise in the interval $[- 5, 5]$.

In this way, the final Dataset 1 (augmented) is six times larger than the original Dataset 1.

#### Train-test split

When validating our method in the previous sections, we performed a cross-validation and patient-stratified scheme. For a fair comparison with the paper^11^, where a static split is performed, in this section, we carry out the same division. Basically, the train set is composed of the spectra coming from patients 1, 3, 5, 6, 8, 10 while the remaining spectra compose the test set (31, 24 and 99 spectra respectively for class EC, CS G1 and CS G2, G3).

#### Results

We point out that, having followed the same procedure of data augmentation and train-test partitioning as in the paper^11^, the results obtained in this section are comparable in all respects to those obtained by CLARA. The only difference lies in the random seed used for data augmentation, but we can assume this difference is negligible. Table 16 shows the results achieved in this setting. The accuracy has improved significantly, and these results are in line with, if not slightly better than, those obtained in the bibliography^11^.

**Table 16:** Results with data augmentation, 3 labels and a static train-test split.

Homology	Accuracy	Vectorization	Classifier
$H_0$	0.974	PS	SVC
$H_1$	0.942	BC	SVC
$H_0+H_1$ (fused)	0.972	PS	SVC
$H_0+H_1$ (concat)	0.975	PS	SVC

### Final test with new data

**Figure 7:**
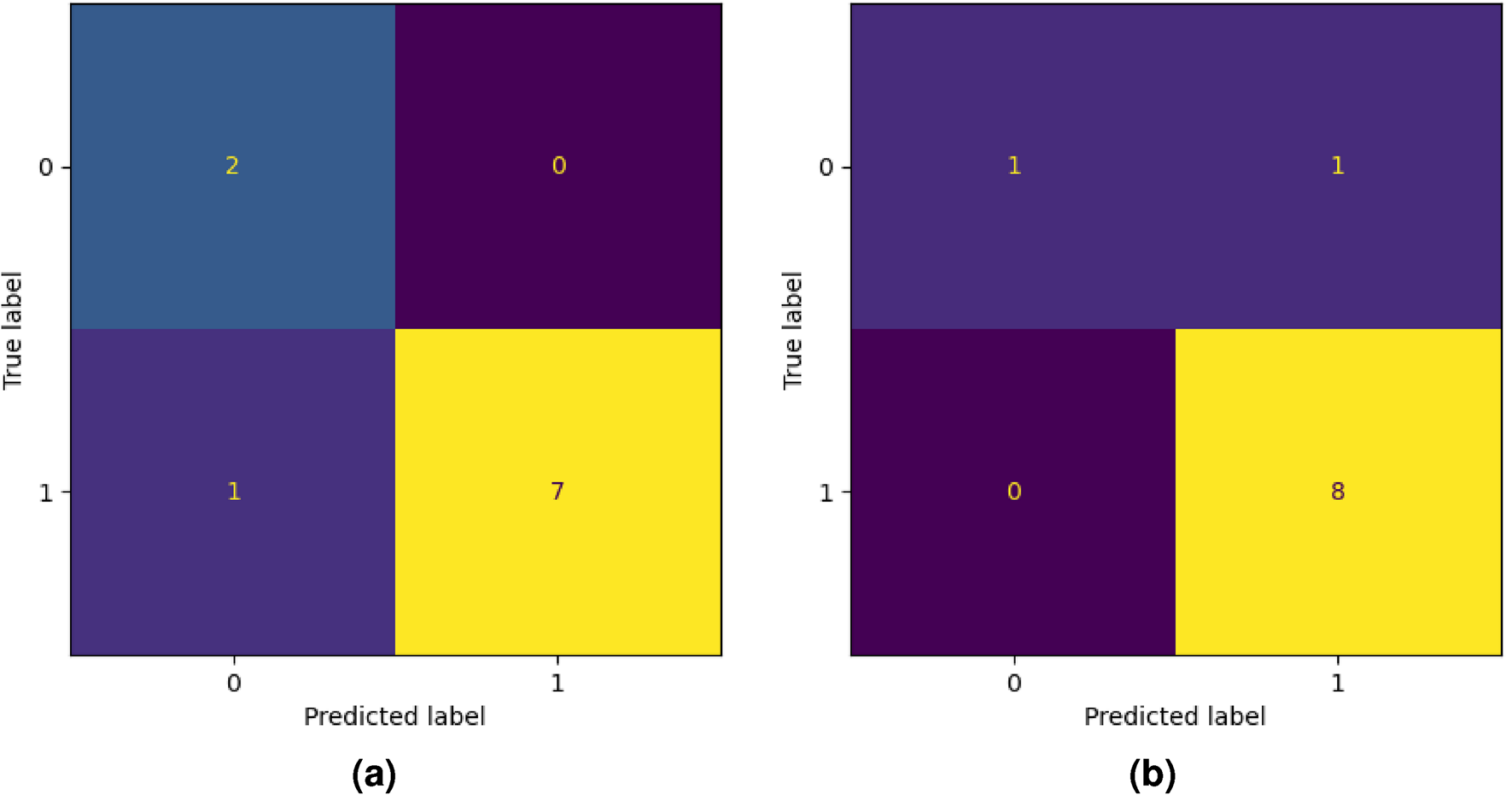
Confusion matrices for binary classification EC *vs.* CS of the best classifier coming from “LOPO validation 2 labels (EC vs. CS)” section (**a**) and the confusion matrix from the best classifier trained on all Dataset 1 (**b**).

**Figure 8:**
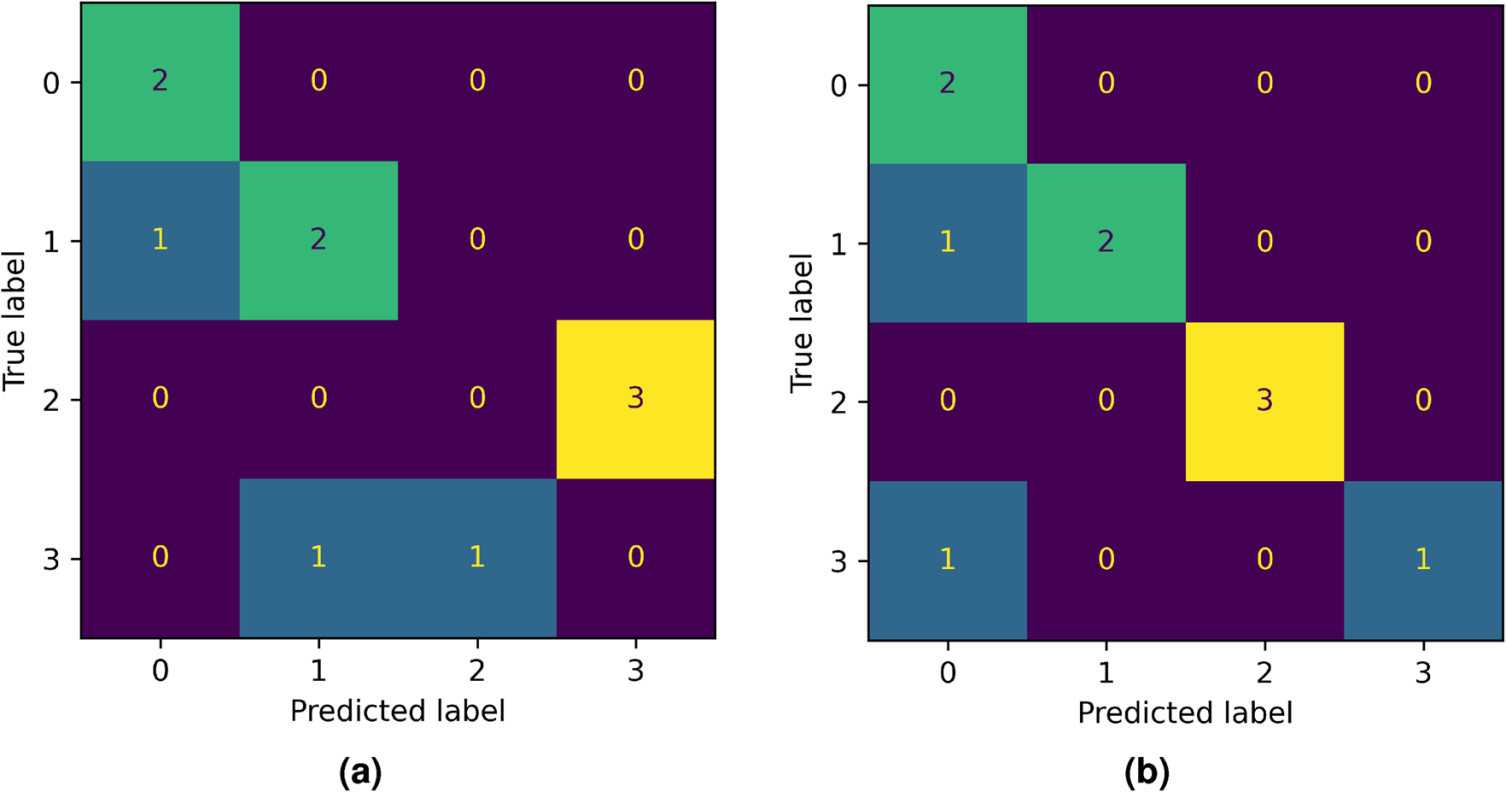
Confusion matrices with 4 labels predicted by the best classifier coming from “LOPO validation 4 labels” section (**a**) and the confusion matrix from the best classifier trained on all Dataset 1 (**b**).

Let us now describe the last type of experiments we performed. In order to assess the generalization capability of the best-performing classification models found through the experimentation described above, we tested the models on new data: Dataset 2. This dataset consists of 10 Raman spectra, one for each patient. Moreover, the acquisition of these spectra occurred at a different time than Dataset 1, with a different equipment. Hence, achieving good results on this dataset would show a very high capability of our model in generalization. Finally, we highlight the fact that there is no intersection between the patients in Dataset 1 with those in Dataset 2. The correct labels of Dataset 2 are [1, 2, 2, 1, 1, 2, 0, 3, 3, 0]. In a EC *vs.* CS classification, this translates in the labels [1, 1, 1, 1, 1, 1, 0, 1, 1, 0]. As a first approach, we directly employed the best classifier coming from “LOPO validation 2 labels (EC vs. CS)” section to classify Dataset 2, resulting in the predicted labels [0, 1, 1, 1, 1, 1, 0, 1, 1, 0]. The accuracy is $90\%$, but the presence of false negatives is discouraging. Repeating the same experiment but retraining the classifiers on the entire Dataset 1, i.e., without a LOPO validation scheme, yields the labels [1, 1, 1, 1, 1, 1, 0, 1, 1, 1]. The accuracy is the same, but there are no false negatives, so it is definitely an improvement. Figure 7 shows the confusion matrices of both these experiments. As a comparison, we repeated these two procedures in the 4 label classification. The classifier trained with a LOPO validation performed poorly, predicting the labels [0, 3, 3, 1, 1, 3, 0, 2, 1, 0], which results in an accuracy of 40%. We highlight that the misclassifications are in adjacent classes, except in one case. This is in agreement with what occurred in binary classification. Training on the entire Dataset 1 yields the labels [0, 2, 2, 1, 1, 2, 0, 0, 3, 0], which corresponds to an accuracy of 80%. The improvement is remarkable and, together with the limitations of the dataset, shows the potential of this method in large-scale applicability. Figure 8 reports the confusion matrices for both of these experiments. Finally, following the great improvement achieved with data augmentation in “Data augmentation” section, we tried to train the classifiers on Dataset 1 (augmented) and test on Dataset 2, but the results were the same. Also, performing the same data augmentation described in “Data augmentation” section on Dataset 2 basically did not change the results, as can be seen in Table 17.

**Table 17:** Results with data augmentation on Dataset 1 and Dataset 2 and 3 labels.

Homology	Accuracy	Vectorization	Classifier
$H_0$	0.783	BC	SVC
$H_1$	0.667	BC	SVC
$H_0+H_1$ (fused)	0.717	PS	SVC
$H_0+H_1$ (concat)	0.700	PL	SVC

## Discussion and conclusions

The present work is devoted to highlighting the potential of combining topological data analysis and machine learning in the very challenging scenario of biochemical understanding of cancer grading through Raman spectroscopy. In order to better appreciate the contribution of the combination of TDA and ML in the proposed pipeline, we performed two ablation studies:

- Supervised learning using the Raman spectra as vectors, fed to the ML part of the pipeline;
- Unsupervised clustering of the vectorizations of the topological PDs (Agglomerative clustering, BIRCH, DBSCAN, K-Means, Mini batch K-Means, Mean-shift, OPTICS, Spectral clustering, and Gaussian mixtures).

Regarding the first study, results show that using the TDA descriptors is much more convenient (for both accuracy and significance) than classifying by the Raman spectra as vectors, in all the classification tasks (4, 3 and 2 labels). On the other hand, even when replacing the ML part of the pipeline with a standard clustering, we noted that the best-performing clustering method is affinity propagation, which has accuracy performances which are only slightly better than those achieved by the complete pipeline. Also, results are very promising with respect to the state of the art, as the classification accuracy outperforms the best results reported in literature^11^, as shown in “Comparison with CLARA^11^” section. Due to the size of the dataset, our results should be considered preliminary but, thanks to the strict validation scheme used, significant. Indeed, the classification accuracy proves to be excellent also when the pipeline is applied to Dataset 2, which is made of never-seen data acquired with a hardware device different from the one used to acquire Dataset 1. Moreover, in every experiments even a slight widening of the training dataset results in a great improvement in the accuracy and specificity (e.g. training on the whole Dataset 1 instead of a LOPO validation), showing excellent potential, both to classify new data and to be vendor neutral (with respect to the RS acquisition). Regarding the applicability of our approach, the proposed pipeline provides a classification model that can be easily integrated into a workflow (as already done in the commercial workstation as for the preprocessing modules), enabling the reduction of time and cost of the grading of cancerous tissues. In conclusion, we are convinced that topological machine learning methods are able to support the classification of data from Raman spectroscopy; also, we plan to perform more conclusive experimentation in the near future, in order to improve not only the classification accuracy of the proposed pipeline, but more importantly to increase the stability of the results with respect to the choice of the best combination {vectorization method, classifier}.

## Data Availability

The request for datasets, both raw and processed data, generated during the present study can be agreed and made directly to the corresponding author.

## References

[1] Short M (2006). Changes in nuclei and peritumoral collagen within nodular basal cell carcinomas via confocal micro-Raman spectroscopy. J. Biomed. Opt..

[2] Long D (2002). The Raman Effect.

[3] Bergholt M (2010). Raman endoscopy for in vivo differentiation between benign and malignant ulcers in the stomach. Analyst.

[4] Bergholt M (2013). Raman endoscopy for objective diagnosis for early cancer in the gastrointestinal system. J. Gastroint. Dig. Syst..

[5] Kong K, Kendall C, Stone N, Notingher I (2015). Raman spectroscopy for medical diagnostics-from in vitro biofluid assays to in-vivo cancer detection. Adv. Drug Deliv. Rev..

[6] Culha M (2015). Raman spectroscopy for cancer diagnosis: How far have we come?. Bioanalysis.

[7] Rau J (2016). Raman spectroscopy imaging improves the diagnosis of papillary thyroid carcinoma. Sci. Rep..

[8] D’Acunto M, Gaeta R, Capanna R, Franchi A (2020). Contribution of Raman spectroscopy to diagnosis and grading of chondrogenic tumors. Sci. Rep..

[9] Savci-Heijink CD, Cleven AH, Bovée JV (2022). Benign and low-grade cartilaginous tumors: An update on differential diagnosis. Diagn. Histopathol..

[10] Suster D, Hung YP, Nielsen GP (2020). Differential diagnosis of cartilaginous lesions of bone. Arch. Pathol. Lab. Med..

[11] Manganelli Conforti P, D’Acunto M, Russo P (2022). Deep learning for chondrogenic tumor classification through wavelet transform of Raman spectra. Sensors.

[12] Saggar M (2018). Towards a new approach to reveal dynamical organization of the brain using topological data analysis. Nat. Commun..

[13] Nielson JL (2017). Uncovering precision phenotype-biomarker associations in traumatic brain injury using topological data analysis. PLoS ONE.

[14] Biscio CA, Møller J (2019). The accumulated persistence function, a new useful functional summary statistic for topological data analysis, with a view to brain artery trees and spatial point process applications. J. Comput. Graph. Stat..

[15] Rabadán R (2020). Identification of relevant genetic alterations in cancer using topological data analysis. Nat. Commun..

[16] Nicolau M, Levine AJ, Carlsson G (2011). Topology based data analysis identifies a subgroup of breast cancers with a unique mutational profile and excellent survival. Proc. Natl. Acad. Sci..

[17] Rucco, M. *et al.* Using topological data analysis for diagnosis pulmonary embolism. Preprint at http://arxiv.org/abs/1409.5020 (2014).

[18] Nielson JL (2015). Topological data analysis for discovery in preclinical spinal cord injury and traumatic brain injury. Nat. Commun..

[19] Conti F, Moroni D, Pascali MA (2022). A topological machine learning pipeline for classification. Mathematics.

[20] Verri A, Uras C, Frosini P, Ferri M (1993). On the use of size functions for shape analysis. Biol. Cybern..

[21] Carlsson G (2009). Topology and data. Bull. Am. Math. Soc..

[22] Adams H (2017). Persistence images: A stable vector representation of persistent homology. J. Mach. Learn. Res..

[23] Bubenik P (2015). Statistical topological data analysis using persistence landscapes. J. Mach. Learn. Res..

[24] Chazal, F., Fasy, B. T., Lecci, F., Rinaldo, A. & Wasserman, L. Stochastic convergence of persistence landscapes and silhouettes. In *Proc. Thirtieth Annual Symposium on Computational Geometry* 474–483 (2014).

[25] Umeda Y (2017). Time series classification via topological data analysis. Inf. Media Technol..

[26] Gholizadeh, S. & Zadrozny, W. *A Tutorial on Topological Data Analysis in Text Mining*. http://bigdataieee.org/BigData2020/files/IEEE_BigData_2020_Tutorial5_TDA_Tutorial.pdf (2020) (Accessed 6 February 2023).

[27] Carlsson E, Carlsson G, De Silva V (2006). An algebraic topological method for feature identification. Int. J. Comput. Geom. Appl..

[28] Zheng, X., Mak, S. & Xie, Y. Online high-dimensional change-point detection using topological data analysis. Preprint at http://arxiv.org/abs/2103.00117 (2021).

[29] Tralie C, Saul N (2018). Ripser.py: A lean persistent homology library for python. J. Open Source Softw..

[30] Cortes C, Vapnik V (1995). Support-vector networks. Mach. Learn..

[31] Breiman L (2001). Random forests. Mach. Learn..

[32] Hoerl AE, Kennard RW (1970). Ridge regression: Biased estimation for nonorthogonal problems. Technometrics.

[33] Pedregosa F (2011). Scikit-learn: Machine learning in Python. J. Mach. Learn. Res..

[34] Allen DM (1974). The relationship between variable selection and data augmentation and a method for prediction. Technometrics.

[35] Frey BJ, Dueck D (2007). Clustering by passing messages between data points. Science.

[36] Davidson I, Ravi SS, Jorge AM, Torgo L, Brazdil P, Camacho R, Gama J (2005). Agglomerative hierarchical clustering with constraints: Theoretical and empirical results. Knowledge Discovery in Databases: PKDD 2005.

[37] Zhang, T., Ramakrishnan, R. & Livny, M. Birch: An efficient data clustering method for very large databases. In *Proc. ACM SIGMOD Intl. Conference on Management of Data (SIGMOD)* 103–114 (1996).

[38] Ester, M., Kriegel, H.-P., Sander, J. & Xu, X. A density-based algorithm for discovering clusters in large spatial databases with noise. In *KDD* (1996).

[39] Hartigan JA, Wong MA (1979). Algorithm as 136: A k-means clustering algorithm. J. R. Stat. Soc..

[40] Sculley, D. Web-scale k-means clustering. In *Proc. 19th International Conference on World Wide Web, WWW 10* 1177–1178. 10.1145/1772690.1772862 (Association for Computing Machinery, 2010).

[41] Comaniciu D, Meer P (2002). Mean shift: A robust approach toward feature space analysis. IEEE Trans. Pattern Anal. Mach. Intell..

[42] Ankerst, M., Breunig, M. M., Kriegel, H.-P. & Sander, J. Optics: Ordering points to identify the clustering structure. In *Proc. 1999 ACM SIGMOD International Conference on Management of Data, SIGMOD’99* 49–60. 10.1145/304182.304187 (Association for Computing Machinery, 1999).

[43] Shi J, Malik J (2000). Normalized cuts and image segmentation. IEEE Trans. Pattern Anal. Mach. Intell..

[44] Dempster AP, Laird NM, Rubin DB (1977). Maximum likelihood from incomplete data via the em algorithm. J. R. Stat. Soc. Ser. B.

